# Response to: “A somatization comorbidity phenotype impacts response to therapy in rheumatoid arthritis: post hoc results from the certolizumab pegol phase 4 PREDICT trial”

**DOI:** 10.1186/s13075-019-1848-4

**Published:** 2019-02-20

**Authors:** Annelise Madison, Barbara L. Andersen, Ali Ajam

**Affiliations:** 1 Institute for Behavioral Medicine Research, The Ohio State University College of Medicine, 460 Medical Center Drive, Columbus, OH 43210 USA; 2 Department of Psychology, The Ohio State University, 1835 Neil Avenue, Columbus, 43210 OH USA; 3 Division of Rheumatology and Immunology, The Ohio State University College of Medicine, 480 Medicine Center Drive, Columbus, 43210 OH USA

Curtis et al. report a post hoc analysis of data from the certolizumab pegol phase 4 clinical trial among rheumatoid arthritis patients [1], p. 4. They infer a “somatization comorbid phenotype” (SCP) among patients who, at intake, had (a) diagnoses of depression, anxiety, or neuropathic pain or (b) were receiving medication for such conditions. Notably, 43% of patients met the criteria. As the authors predict, these individuals were more likely to drop out and report adverse side effects and less likely to achieve low disease activity status. They conclude that SCP may mask the drug’s true effectiveness, and thus, they question whether to “include, identify, or stratify” these patients in future clinical trials. Their operationalization of SCP and resulting conclusion are seriously flawed.

First, the authors’ definition of SCP does not index somatization, or patient-reported physical symptoms with no known organic cause. Medically unexplained somatic complaints and depression/anxiety are not inherently comorbid. In this study, SCP-positive patients did not somaticize: although their clinician-rated disease activity was greater than SCP-negative individuals, they did not report greater symptom severity. Therefore, the authors’ SCP grouping lacks construct validity. Disregarding the SCP label, their findings replicate prior research that links depression and anxiety with greater disease activity in RA. This study only muddied the waters by grouping dissimilar individuals and inaccurately labeling them as somaticizers.

Most troubling are the authors’ three recommendations to eliminate this unwanted variance among RA patients: exclude, label, or stratify these patients. Stratification is unnecessary, as these patients comprise almost half of the population of interest and groups would be equated on this dimension if a sufficiently large sample is randomized. Identifying depressed and anxious RA patients is the only viable option they suggest—but only if the purpose is to offer supportive treatment. Excluding these patients is untenable from a biological perspective. A depressive episode increases risk for autoimmune disease onset [2] and fuels inflammation [3], while RA-related inflammation can maintain and even worsen depression [4]. An RA sample free of depression/anxiety would not represent the RA population and treatment efficacy would be artificially inflated.

There are many troubling aspects to Curtis et al.’s (2017) report. It offers no contribution to the existing literature, but instead pejoratively labels RA patients struggling with mood dysregulation and/or neuropathic pain. The final recommendations for managing these patients via research design strategies harken to a dangerous and unethical view of individual differences.
