# Supplementary material for: Response to: “A somatization comorbidity phenotype impacts response to therapy in rheumatoid arthritis: post hoc results from the certolizumab pegol phase 4 PREDICT trial”
Source: Arthritis Res Ther. 2019 Feb 20;21:65. doi: 10.1186/s13075-019-1848-4 (PMC6381628; doi:10.1186/s13075-019-1848-4)
Supplement: Related texts

# Reply: Authors’ response

**Authors:** Jeffrey R. Curtis, Christopher Herrem, Matladi N. Ndlovu, Cathy O’Brien, Yusuf Yazici

We appreciate the opportunity to respond to the misapprehensions raised by Madison et al. of our analysis. They offer critique based on a fundamental misunderstanding of both the context and intent behind our approach, which should be viewed from the perspective of the design of a rheumatoid arthritis (RA) clinical trial, not guidance for patient-centric clinical care. We share their implied assertion that the experiences of all people living with RA, including the many who also face depression and anxiety, ought to be reflected in the study of the effectiveness and safety of RA treatments.

That said, the assertion by Madison and colleagues that depression, anxiety, and medically unexplained somatic complaints that result in chronic pain “are not inherently comorbid” seems to inappropriately minimize the importance that these conditions have on the lives of RA patients. Moreover, failure to recognize them as distinct from active RA potentially limits the opportunity to address and treat them independently. Secondly, the statement that patients with the SCP phenotype did not report greater symptom severity ignores the evidence we provided in the manuscript. Baseline pain, patient global, and tender joint count were numerically higher in SCP patients. We also showed that the benefits of starting certolizumab pegol were attenuated by approximately 10–15% for the key trial outcomes (e.g., low disease activity, ACR 20/50/70). Since patient-derived assessments factor importantly in these composite RA outcomes, their statement that outcome differences were solely driven by clinician ratings seems unsupported by evidence from our study.

Additionally, Madison et al. criticized our grouping of somewhat distinct conditions into a SCP phenotype. RA trials typically desire to minimize participant burden and avoid “non-essential” data collection. Thus, information about depression, anxiety, and comorbidities associated with non-RA pain (e.g., diabetic neuropathy, spondylosis, fibromyalgia) is often lacking. For the purposes of this analysis, information classifying patients by SCP status was from the medical history and concomitant medication data collected at baseline. Prospectively collecting these data is generally not a priority in trials and, once the trial has ended, the opportunity is lost. Thus, our phenotype offers a practical approach that can be applied even retrospectively to already-collected data to identify patients who may have an attenuated response to an effective RA treatment. Indeed, we have recently shown that this concept translates to other data sources (e.g., an EHR-based registry), where concomitant fibromyalgia resulted in an attenuated treatment response following initiation of a new RA medication [5].

They find fault with our recommendation to consider the possibility of accounting for the SCP phenotype in the design of a trial. While it is true that randomization would balance SCP+ patients between treatment groups in large samples, and thus suggest that “stratification is unnecessary,” they neglect the fact that one cannot randomize patients to the SCP phenotype, and not all RA studies are randomized. Indeed, within an observational study, identifying individuals with the SCP phenotype (or its components) allows one to adjust for or otherwise control for this factor. Moreover, even with randomization, rheumatologists place high importance on not only the relative effect of RA medications versus a comparator treatment, but also on the absolute response (the “60-40-20” ACR response) [6]. Because the SCP phenotype meaningfully reduces absolute response rates by 10–15%, including large numbers of patients with this phenotype in a RA therapy trial will inappropriately penalize that therapy’s perceived effectiveness. Data from international studies comparing the prevalence of some comorbidities that make up the SCP phenotype (e.g., depression) have shown high variability between countries [7]. Therefore, the magnitude of the effect on RA outcomes may be substantial in clinical studies where the prevalence of SCP phenotype conditions varies widely. As for another possibility we mentioned, excluding patients with the SCP phenotype, we agree that this would excessively limit generalizability and likely would be inadvisable for most trials.

Finally, we are disappointed that Madison et al. feel that identifying mood dysregulation and/or neuropathic pain in an RA patient assigns them a “pejorative label.” We respectfully disagree and would offer the perspective that rheumatologists can and should be concerned with a holistic and personalized approach to RA patient management. As part of high-quality clinical care, we believe that depression, anxiety, and non-RA-related causes of pain need to be identified and treated. However, this does not always entail changing biologic therapy nor deeming a patient’s RA treatment to be ineffective. The analysis we presented quantifies the extent to which SCP-related conditions may moderate the measured effectiveness of an anti-TNF in the context of a RA clinical trial. While many RA trials are not intended to mirror clinical practice, we nevertheless strongly agree that RA patients’ individual differences must be considered and valued in both research and routine practice settings. A growing number of examples exist within rheumatology where patient-centered research is at the forefront of scientific questions that are being asked and answered [8].
